# Developing a Cost-Effective Surgical Scheduling System Applying Lean Thinking and Toyota’s Methods for Surgery-Related Big Data for Improved Data Use in Hospitals: User-Centered Design Approach

**DOI:** 10.2196/52185

**Published:** 2024-05-24

**Authors:** Chien-Chung Lin, Jian-Hong Shen, Shu-Fang Chen, Hung-Ming Chen, Hung-Meng Huang

**Affiliations:** 1 Department of Orthopedic Surgery Taipei City Hospital Taipei Taiwan; 2 General Education Center University of Taipei Taipei Taiwan; 3 Department of Finance Chihlee University of Technology New Taipei City Taiwan; 4 Department of General Surgery Taipei City Hospital Taipei Taiwan; 5 Department of Otorhinolaryngology Taipei City Hospital Taipei Taiwan

**Keywords:** algorithm, process, computational thinking, continuous improvement, customer needs, lean principles, problem solving, Toyota Production System, value stream map, need, needs, operating room

## Abstract

**Background:**

Surgical scheduling is pivotal in managing daily surgical sequences, impacting patient experience and hospital resources significantly. With operating rooms costing approximately US $36 per minute, efficient scheduling is vital. However, global practices in surgical scheduling vary, largely due to challenges in predicting individual surgeon times for diverse patient conditions. Inspired by the Toyota Production System’s efficiency in addressing similar logistical challenges, we applied its principles as detailed in the book “Lean Thinking” by Womack and Jones, which identifies processes that do not meet customer needs as wasteful. This insight is critical in health care, where waste can compromise patient safety and medical quality.

**Objective:**

This study aims to use lean thinking and Toyota methods to develop a more efficient surgical scheduling system that better aligns with user needs without additional financial burdens.

**Methods:**

We implemented the 5 principles of the Toyota system: specifying value, identifying the value stream, enabling flow, establishing pull, and pursuing perfection. Value was defined in terms of meeting the customer’s needs, which in this context involved developing a responsive and efficient scheduling system. Our approach included 2 subsystems: one handling presurgery patient data and another for intraoperative and postoperative data. We identified inefficiencies in the presurgery data subsystem and responded by creating a comprehensive value stream map of the surgical process. We developed 2 Excel (Microsoft Corporation) macros using Visual Basic for Applications. The first calculated average surgery times from intra- or postoperative historic data, while the second estimated surgery durations and generated concise, visually engaging scheduling reports from presurgery data. We assessed the effectiveness of the new system by comparing task completion times and user satisfaction between the old and new systems.

**Results:**

The implementation of the revised scheduling system significantly reduced the overall scheduling time from 301 seconds to 261 seconds (*P*=.02), with significant time reductions in the revised process from 99 seconds to 62 seconds (*P*<.001). Despite these improvements, approximately 21% of nurses preferred the older system for its familiarity. The new system protects patient data privacy and streamlines schedule dissemination through a secure LINE group (LY Corp), ensuring seamless flow. The design of the system allows for real-time updates and has been effectively monitoring surgical durations daily for over 3 years. The “pull” principle was demonstrated when an unplanned software issue prompted immediate, user-led troubleshooting, enhancing system reliability. Continuous improvement efforts are ongoing, except for the preoperative patient confirmation step, which requires further enhancement to ensure optimal patient safety.

**Conclusions:**

Lean principles and Toyota’s methods, combined with computer programming, can revitalize surgical scheduling processes. They offer effective solutions for surgical scheduling challenges and enable the creation of a novel surgical scheduling system without incurring additional costs.

## Introduction

The operating room (OR) is a large department within a hospital serving patients. The scheduling of surgeries determines the daily order in which patients undergo surgery. Most patients prefer to undergo surgery as early as possible, as waiting for surgery is both mentally and physically torturous, not to mention the hunger from fasting. If the number of surgeries scheduled or the total time required exceeds the capacity of the OR for a day, some surgeries may be postponed. Any delay in a patient’s operation will cause patient dissatisfaction with the hospital and the surgeon or confrontation between surgical units and personnel. OR nurses do not know how many hours it will take to complete all the operations on a given day, how much manpower will be needed, and whether they will need to work overtime. Surgeons also want to know how long patients must wait before they are moved to the OR. According to analysis, the main cause of this issue is the lack of a surgical scheduling system that can meet the needs of users. Uncertainty creates panic. When the IT department was asked to provide a surgical scheduling system that could meet the needs of OR staff and surgeons in our hospital, they could not provide such a system.

Why did the IT department have no ability to provide us with a surgical scheduling system that could meet our needs? A literature review of surgical scheduling identified 300 studies published before 2018; however, no widely accepted surgical scheduling method has been developed [1]. The reasons for this can be attributed to the many uncertainties related to scheduling. One of the main challenges is predicting the time each surgeon requires for various surgeries on patients with different conditions [1]. Even when patients have the same disease, their bodies and disease severities differ, the site and scope of the surgery also differ, and many factors, such as different surgeons, make it difficult to predict surgery times.

ORs incur a cost of approximately US $36 per minute, making them one of the most expensive departments in a hospital [2]. Without a surgical scheduling system, it is impossible to efficiently manage the use of OR personnel, equipment, and instruments, impacting patient safety and quality of medical care and increasing the cost of surgeries. These factors represent the real problem that must be solved. Choosing the appropriate approach to deal with a problem is the first step to successfully resolve it. To this end, we have studied the Toyota Production System (TPS) and successfully applied it in clinical operations [3]. TPS is most famous for solving workplace problems; it can provide a good thinking model and effective scientific methods to help solve problems. Since the TPS has spread from Japan to the rest of the world, because of the application and development of lean experts in different fields, it has been given different names, such as lean production, lean manufacturing, and lean management [4-7]. According to the TPS, waste is considered the root of all problems, and there are 7 types of workplace waste [4-7]. In the book “Lean Thinking,” Womack and Jones [4] adopt the essence of the TPS, add their insights to propose 5 principles of lean thinking, and mention an eighth type of waste: waste that does not meet customers’ needs.

Our main problem is that we do not have a surgical scheduling system that meets our needs (ie, having the eighth type of waste). The goal of this paper is to address the OR computer information problems we encounter by using the principles and methods of lean thinking and to create a surgical scheduling system that meets users’ needs within 1 year.

## Methods

### Study Design

Our initial plan was to use the 5 principles of lean thinking to guide our thinking direction and to adopt Toyota’s methods as our tools. The 5 principles involve specifying the value and identifying the value stream, flow, pull, and perfection [4]. Waste is defined as any obstacle to accomplishing a task [4-7]. Womack and Jones [4] apply the concept of streams to embody each valuable step in accomplishing a task and use flow to run through all the valuable steps, thus forming a value stream map (VSM).

The first principle is to specify value. In everyday life, the value of an object, product, or service is determined by the customer or user, even though producers create the value itself. The primary role of value is to fulfill customers’ needs [4-7]. The concept of value can be compared to a target in archery. Any hindrance in identifying or reaching the target symbolizes waste. Achieving the bullseye, or the red center of the target, means the customer obtains 100% of the value. As the shot deviates farther from the center, the value the customer receives diminishes. The more a product aligns with customer needs—hitting close to the bullseye—the more customers will willingly purchase it. The exploration of value resembles the search for an archery target. Upon finding the target, defining it is tantamount to specifying a value. When we run our hospital’s “operation scheduling query system,” we can see that each patient’s data in the “operation scheduling query system” comprises 41 fields. If there are 50 patients undergoing surgery on a given day, there are at least 2050 fields of data to review. In this system, the output report shown on the computer screen (Microsoft Excel 2010 extension filename.xls) can present data for approximately 26 patients (rows) and 18 fields (columns). Finding the data of interest is difficult, and there is almost no way to read all patient information on 1 screen. If the data within the “operation scheduling query system” are printed out, each piece of A4 paper can only provide information for 5 patients. Thus, 10 sheets of paper are required for 50 patients. Printed information has another shortcoming, as it does not include information about the special surgical instruments needed. Therefore, the “operation scheduling query system” in its current form does not provide substantial value. In the principle of lean thinking, to clearly understand a problem or to break it down in detail, drawing a VSM related to the problem is an indispensable and crucial step.

**Figure 1:**
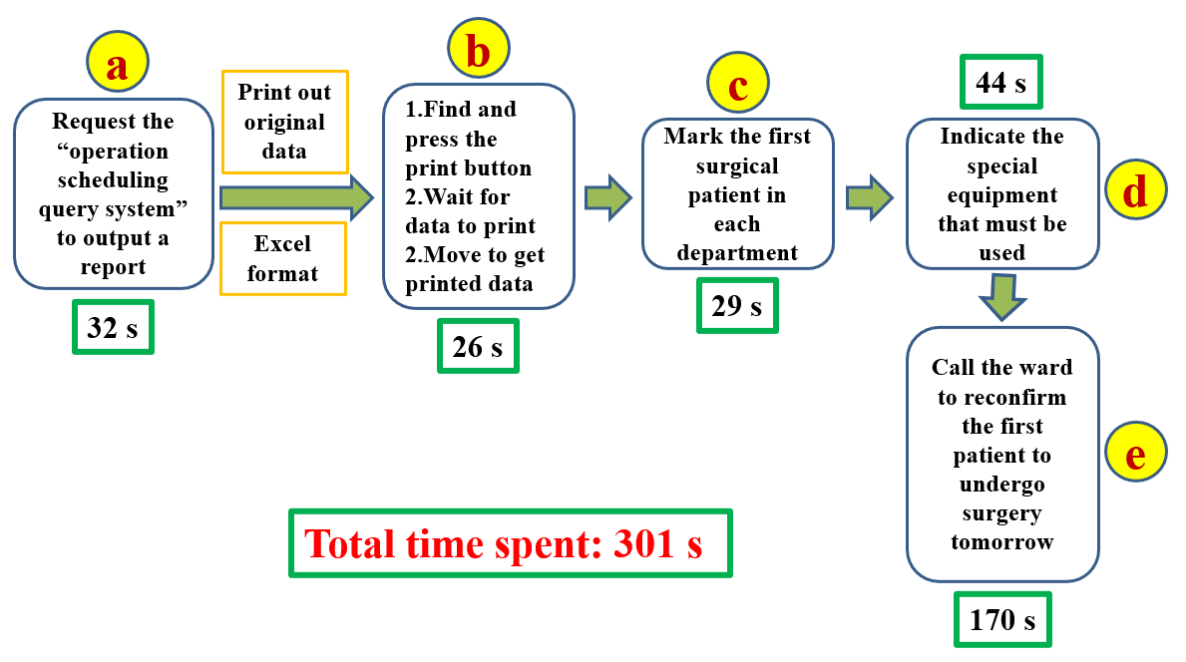
User-centered value stream map before improvement with the time spent on each action. s: seconds.

The second principle is to identify the value stream. The function of a VSM is to provide a means of holistic thinking [4,8]. To draw a VSM, we asked the OR nurses to identify and draw all the steps related to surgery scheduling in an ordinary scheduling process. Since value is defined by users [4], we take a nurse-centered view and preserve the valuable parts of the ordinary scheduling process to formulate the VSM of the current process (Figure 1).

Because the “operation scheduling query system” was difficult to use, we examined the whole system related to the “operation scheduling query system” and found that under the mother system (the hospital’s surgery information system) of the “operation scheduling query system,” there is an “operating room nursing log” subsystem related to patient surgery data. The “operation scheduling query system” records the data of all surgical patients before surgery, and the “operating room nursing log” records the data of all surgical patients during and after surgery. When examining the hospital’s “operating room nursing log” subsystem, we found the subsystem record, in detail, the time patients entered the OR, the start and end time of anesthesia, the time surgeons started and ended an operation, and the time to travel to the anesthesia recovery room, allowing us to calculate the average time spent for each surgery by different surgeons. In the process of solving problems and eliminating waste, we found that the most direct way to handle daily recurring tasks or issues, such as working with computer information systems, is to learn computer programming to leverage the advantages of rapid computer calculations and information processing. One of the countermeasures we formulated was to use the big data from the hospital’s “operating room nursing log” to calculate the average time spent for each surgery by different surgeons. To implement the countermeasure, we studied Excel’s functions and spent half a year learning to code in the Visual Basic for Applications (VBA) programming language within Microsoft Excel 2010 [9]. We used the VBA programming language to write a macro that used approximately 12,000 pieces of patient surgical data from the “operating room nursing log” to calculate the average time spent on different operations performed by different doctors and the average time spent on different anesthesia methods. The average time spent by the surgeon and the anesthesiologist to complete an operation is stored in the “surgeon’s surgical spent time” file.

**Figure 2:**
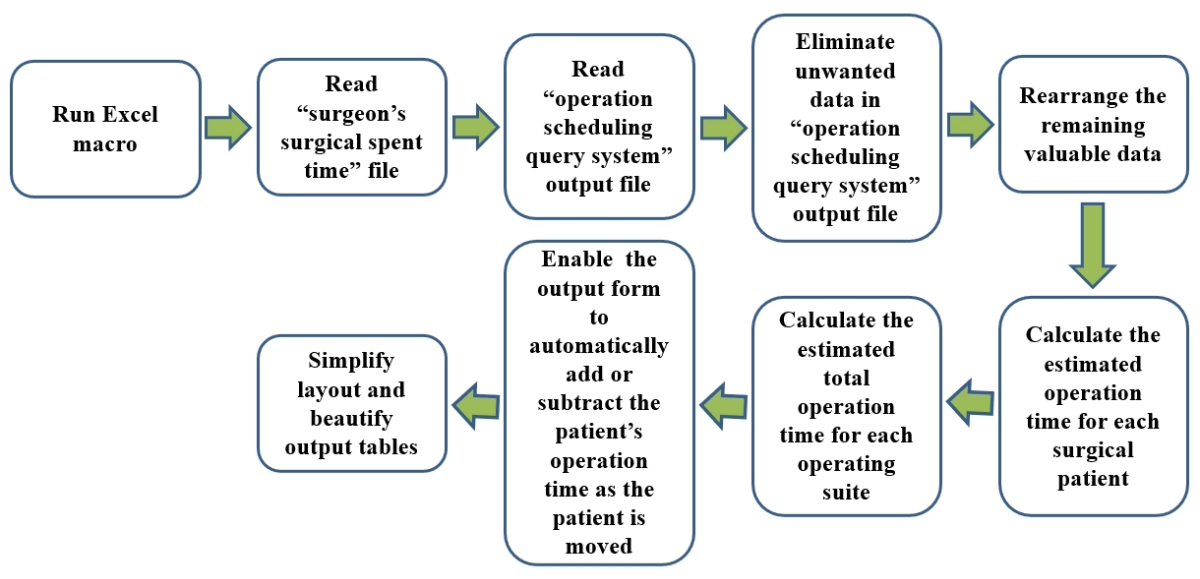
An algorithm before translation into a computer language that can meet the needs of operating room colleagues. The primary function of the algorithm is to filter out unnecessary data and reorganize the remaining valuable information. It calculates the time various surgeons need to perform different types of surgeries and determines the total surgical time required for each operating suite. Finally, the algorithm generates 2 concise and visually appealing report formats in Excel worksheets.

**Figure 3:**
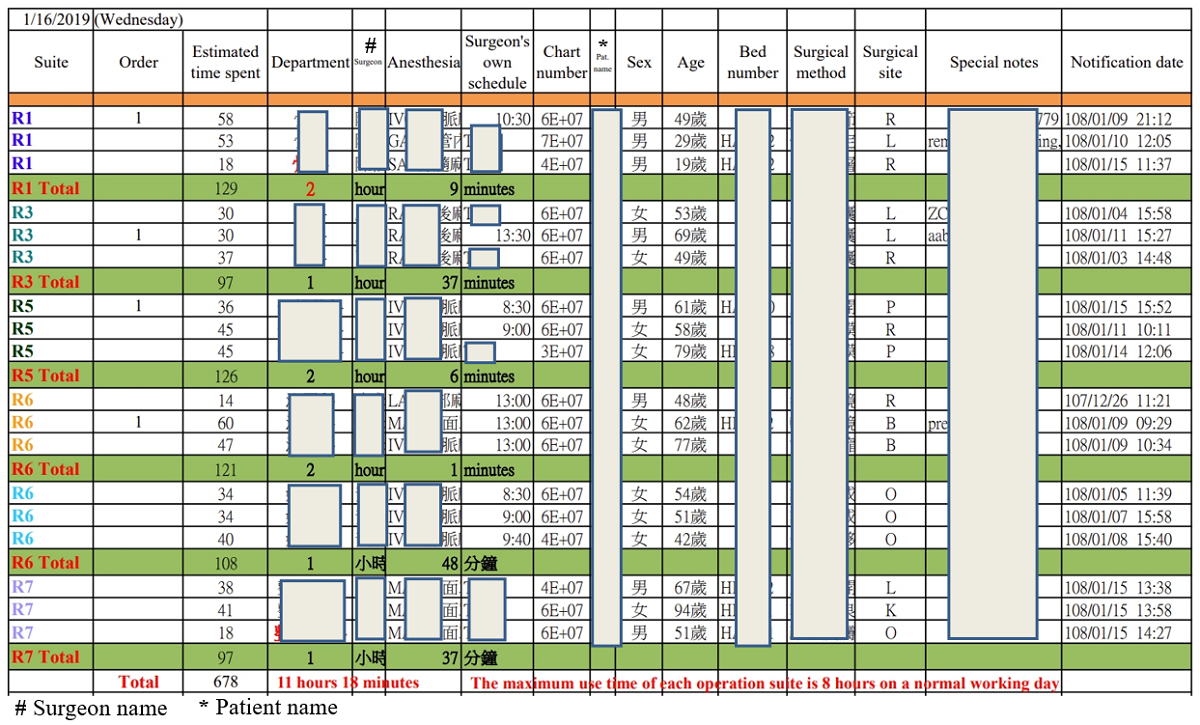
An abbreviated version of the screen output form containing only the 16 most important fields. This form displays the daily usage time for each operation suite and the total estimated time for all operations on that day. Designed to partially obscure patient information for privacy, it is suitable for publication in the LINE group.

**Figure 4:**
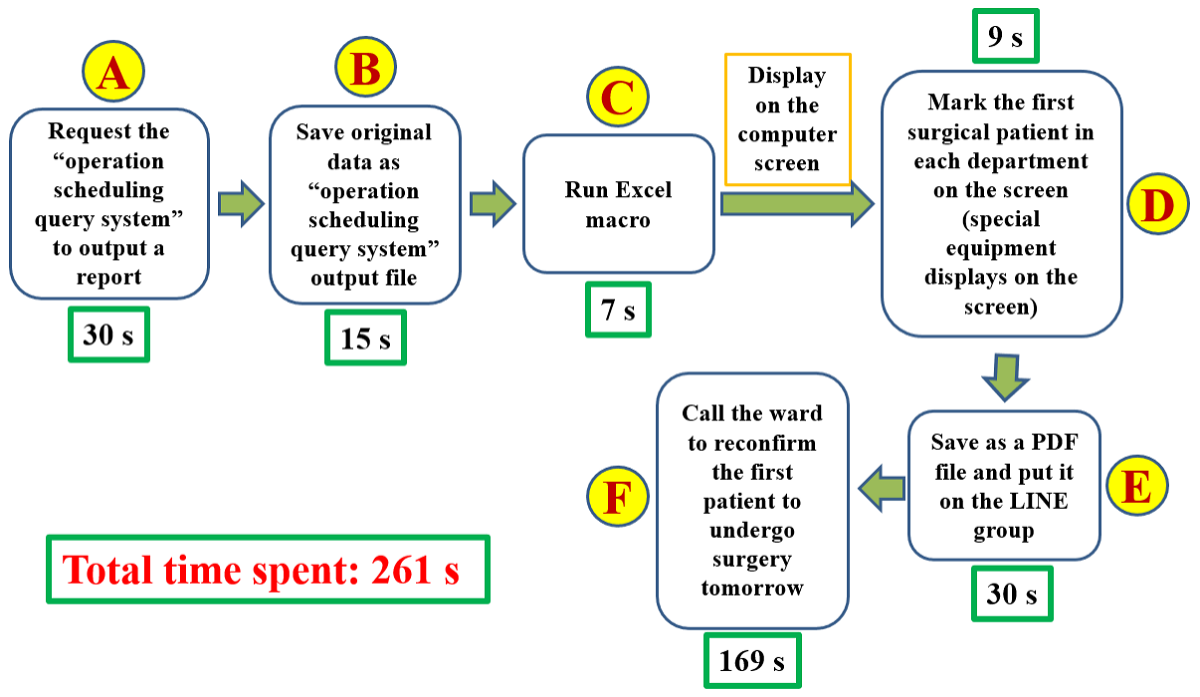
User-centered value stream map after improvement with the time spent on each action. s: seconds.

The other countermeasure developed involved coding another macroprogram using the data from the “surgeon’s surgical spent time” file. This program was designed to estimate the time taken by different surgeons to perform various types of surgery and generate relatively concise screen report formats. We have learned that when training a computer program, it is important to define the algorithm before writing the programming code [10]. We find that thinking about and writing algorithms is similar to constructing VSMs [8-10]. We use the concepts and methods from drawing VSMs to help us complete the surgical scheduling algorithm [9,10]. Figure 2 shows a program algorithm that meets the expectations of colleagues in the OR. Translating an algorithm into programming code is easier and less error-prone than other approaches. We used VBA to translate the algorithm in Figure 2 into a macro, referred to as the “surgery scheduling macro” (hereafter, “Excel macro”). The Excel macroestimates the time taken for different surgeons to perform different types of surgery; that is, the macro can calculate the total surgical time required for patients with different diseases. The Excel macro can extract 16 valuable fields from the original 41 fields of each piece of patient-related data stored in the “operation scheduling query system” output file and generate 2 relatively concise and visually pleasing screen report formats (Excel worksheets). The first format, which partially obscures patient information for privacy, can be published in the LINE group (Figure 3). The second format, containing the patient’s contact number and preferred contact method, is convenient for OR nurses. The screen form, shown in Figure 3, shows how much time each operation suite is in use on a given day and displays the total estimated time for all operations on a given day. The successful development of the Excel macro standardized the VSM of the surgical schedule, as shown in Figure 4.

Although the VSM after improvement (Figure 4) appeared more convenient to implement and saved time, accurate numerical data were required to confirm its function. There were a total of 21 nurses in the OR. After the head nurse and the leader were excluded, a total of 19 OR nurses were included in the comparison test of the VSM before (Figure 1) and after improvement (Figure 4). Each OR nurse conducted surgical scheduling using the VSM before and after improvement. The OR nurse leader recorded the time spent on each step of the VSM before (Multimedia Appendix 1) and after (Multimedia Appendix 2) improvement. Additionally, each participant completed a postoperative questionnaire (Multimedia Appendix 3). Data acquisition and questionnaires were conducted anonymously. The time spent before and after improvement was compared using paired *t* tests (Microsoft Excel 2016). A P (2-tailed) <.05 was considered significant. We expected that the percentage of “Yes” answers to each item of the questionnaire survey would exceed 80%.

### Ethical Considerations

This study is part of our OR lean projects, which have been supported and approved by the OR management committee and the hospital director. The Lean project is mandated by the City Government for its public hospitals. According to local legislation and institutional requirements, ethical review and approval were not required for this type of project, which involves analyzing operational procedures without direct intervention on human participants. Please refer to the Personal Data Protection Act [11], Chapter I (General Provisions), Article 6, which outlines exceptions for data collection in the contexts of health care, public health, or crime prevention research, provided such data cannot lead to the identification of specific individuals.

Consistent with national legislation and institutional requirements, written informed consent was not required for this study [12]. For more details, please refer to Multimedia Appendix 4. This document includes an official administrative document released by the government and its English translation. All data were deidentified to ensure privacy and confidentiality. No compensation was provided to participants, as this paper did not involve any direct participation. It is also ensured that no individual participants or users can be identified in any images in this paper or supplementary materials.

## Results

The mean total time spent before improvement was 301 (SD 57.5) seconds (Multimedia Appendix 1), after improvement, it was 261 (SD 45.1) seconds (Multimedia Appendix 2). The variance of the time spent before improvement was 3306, after improvement, it was 2032. The 2-tailed *P* value was .02; thus, the result is significant and sufficient to state that the time spent on the process significantly decreased after the improvement. Focusing only on the parts of the process that have been changed (b, c, and d in Figure 1 and B, C, D, and E in Figure 4), the mean total time spent was 99 (SD 32.1) seconds before improvement and 62 (SD 20.5) seconds after. The variance of the time spent was 1032 before improvement and 420 after. The *P* value (2-tailed) was <.001; thus, the result is significant (*P*<.001) and sufficient to state that the time spent on the changed processes was decreased significantly. Multimedia Appendix 3 shows that 84% (16 out of 19 participants) of OR nurses chose to use the process after improvement. However, 21% (4 out of 19 participants) of OR nurses thought they were more familiar with the original process and did not think the improved process was easier to use. The surgical scheduling form, with sensitive patient data hidden, is displayed in the LINE group immediately after the Excel macroruns. Under normal circumstances, there is no need to print out any paper.

The third principle is flow. Flow production means that a machine is arranged according to the processing order and product production is not interrupted and does not become stagnant during manufacturing. The Excel macro is user-friendly; pressing a large button in the Excel worksheet produces 2 forms within 2 seconds that meet the needs of OR staff. The Excel macro also stores the file name and execution date, hours, minutes, and seconds; thus, 2 files will never have the same name. A new form is generated using the macro if a new patient is added to the schedule. The program can be executed at any time. The VSM in Figure 4 and the macro have now been used daily for over 3 years and can be executed whenever needed, and no problems have arisen to date. During this period of use, we identified an additional added value: the macro can be used to monitor the reasonable total number of operations and the total operation time per surgeon per day and monitor how many nonemergency patients receive surgery on the day of admission.

The fourth principle is pull. We developed the Excel macro and changed the VSM, both of which are mainly pulled by the needs and expectations of relevant colleagues. Last year, the hospital’s Microsoft Excel was upgraded from the 2010 version to the 2016 version (operating system: Windows 10), which caused errors in the execution of the program, and the program was unavailable for 2 months. Individuals felt inconvenienced during this period. Their needs and expectations prompted us to study the newer VBA language and modify the program to restore its function.

The fifth principle of lean thinking is perfection. Perfection means continuous, never-ending improvement. For over 3 years, our surgical schedule has been announced in the LINE group, which is open and transparent, thus allowing individuals to provide their opinions. The content of the output report has been changed in alignment with the users’ needs. The improved VSM is published in the OR next to the computer and is visible to all staff.

Before the development of our surgical scheduling program, we had conducted an in-depth understanding of user requirements. As a result, for over the 3 years of deploying the program user requirements have remained consistent without a need for updates. However, to ensure continuous improvement we have identified 3 small goals. The first goal is enhancing the protection of doctors’ privacy, the second goal is monitoring a reasonable number of surgeries for doctors, and the third goal is ensuring fairness in surgical scheduling. For instance, to safeguard doctors’ privacy in our publicly accessible surgical scheduling form on LINE, we have replaced doctor names with a coded alphanumeric system. Regular assessment of the effectiveness of this privacy maintenance will be conducted. Based on the assessment, further enhancements will be made to ensure optimal privacy maintenance. Daily monitoring of the number of surgeries scheduled for each surgeon will be implemented on the LINE form. The head of the Department of Surgery will assess the impact on doctors’ workload and well-being. Rescheduling will be carried out as needed to ensure a reasonable number of surgeries for each doctor. The reasonability and fairness of surgical scheduling are also actively monitored daily via the LINE form. Immediate actions will be taken to rectify any unfair scheduling practices. This transparent system serves not only as a means for daily management but also as a mechanism for managing any abnormalities in the OR. Consequently, we have found that OR management has become more streamlined.

To date, the final aspect of the process is the only part that has not been improved, which is “call the ward to reconfirm the first patient to undergo surgery tomorrow” (Figure 4 step F). No changes have been made to this process because confirming the patient’s identity and surgical information individually (in detail) is the most important step in hospital regulations for patient safety.

## Discussion

### Principal Findings

The main finding of this paper was that the integration of lean principles and Toyota’s methods with computer programming can rejuvenate surgical scheduling processes, providing effective solutions to scheduling challenges and facilitating the development of a new surgical scheduling system without incurring extra expenses.

### Value Identification

Although the TPS is typically used in manufacturing, its application in health care has grown [13-15]. It is rare to see TPS used to enhance computer information systems, yet this was our approach when our operation scheduling system fell short of expectations. Familiar with TPS’s problem-solving framework, we adopted lean thinking principles to ensure we addressed the right issues [3]. According to Womack and Jones [4], true value is defined solely by the user’s needs. For us, this meant understanding the specific needs of OR staff. OR nurses needed to predict the duration and staffing for the next day’s surgeries, and surgeons wanted to know their waiting times between surgeries. This user-centered focus guided our system redesign.

### Problem Decomposition

To address the requirements of OR colleagues and surgeons, we needed to accurately calculate the time each surgeon spends on different surgeries. While literature shows that complex mathematical models or simulations are common for this purpose [1,16-18], these methods are impractical and costly for actual OR settings. Grounded in the core TPS belief that solutions should arise from within the workplace [3-5], we used existing data from our “operating room nursing log” to estimate average surgery and anesthesia times. This method, backed by clinical experience, proved reliable as skilled surgeons consistently match historical times, with recalibrations needed only annually for less experienced surgeons.

Our review of the “operation scheduling query system” identified that only 16 of the 41 data fields were necessary, highlighting a significant overproduction of data. This overproduction, a key waste type under TPS, not only complicates data management but also masks other inefficiencies such as inventory excess, unnecessary movements, and processing delays, often compounded by data errors and system bugs [4-6]. Therefore, the most urgent task was to eliminate the waste associated with having too much information.

### Problem-Solving

To tackle data overproduction waste, we crafted an Excel macro that targets 16 critical fields within the “operation scheduling query system,” efficiently streamlining data extraction. This macro, designed using a simple comparison and addition algorithm (Figure 2), calculates daily operating times and OR hours, enhancing report clarity and accuracy. It also corrects departmental mismatches by linking physicians’ names with their departments, thus addressing defect-related waste. With the schedules now published in the LINE group, we have eliminated the need for physical printouts, thus reducing paper, movement, and waiting wastes, allowing nurses to manage all surgical scheduling digitally.

The output report (Figure 3) allows OR managers to verify the appropriateness of surgical loads and timings. It facilitates daily staffing decisions by OR and anesthesiology head nurses based on current schedules. The macro’s flexibility means it can be run anytime to update or verify surgery additions, aiding in operational adaptability. If the OR’s capacity is exceeded, surgeries might be rescheduled—a decision made through consultations involving anesthesiologists, surgeons, patients, and their families, adhering to TPS’s respect for people principle.

### Improved Flow of the VSM

Using our developed macroprograms, we enhanced the hospital’s “operating room nursing log” and “operation scheduling query system,” resulting in a refined VSM (Figure 4). In this map, any disruption signifies obstacles that lead to inefficiencies. Smooth flows in the value stream deliver genuine value to users [4,8]. In programming, organized and error-free code execution is crucial; errors suggest bugs that impede flow, akin to waste in TPS. By focusing on debugging and waste elimination, we achieved a more efficient flow in our processes.

### User Satisfaction

The principle of pull, a cornerstone of lean thinking, dictates that production should only meet immediate market demand [4,5]. This approach ensures that products and services are only created based on actual needs, avoiding overproduction—one of the most severe forms of waste [4-6]. Our surgical scheduling system embodies this principle; the Excel macro we developed produces surgical schedules on-demand, ensuring they are generated in real-time as needed by staff. This system aligns with the just-in-time production philosophy of producing the right product in the right amount at the right time [4-7].

The Excel macro efficiently manages data by filtering out unnecessary information, thus eliminating multiple forms of waste: overproduction, waiting times, movement (since it is used directly at the workstation), and transportation (as reports are electronically distributed via the LINE group). Additionally, there is no need for a physical inventory of reports, minimizing inventory waste. Accuracy checks further reduce the risk of errors in outputs. Privacy has been a priority, with reports saved as PDF files before sharing, enhancing transparency and supporting fair and collaborative surgical scheduling. This method reflects the TPS’s Kanban system [4-7], promoting effective visual management and fostering improved interprofessional relations.

### Continuous Improvement

**Figure 5:**
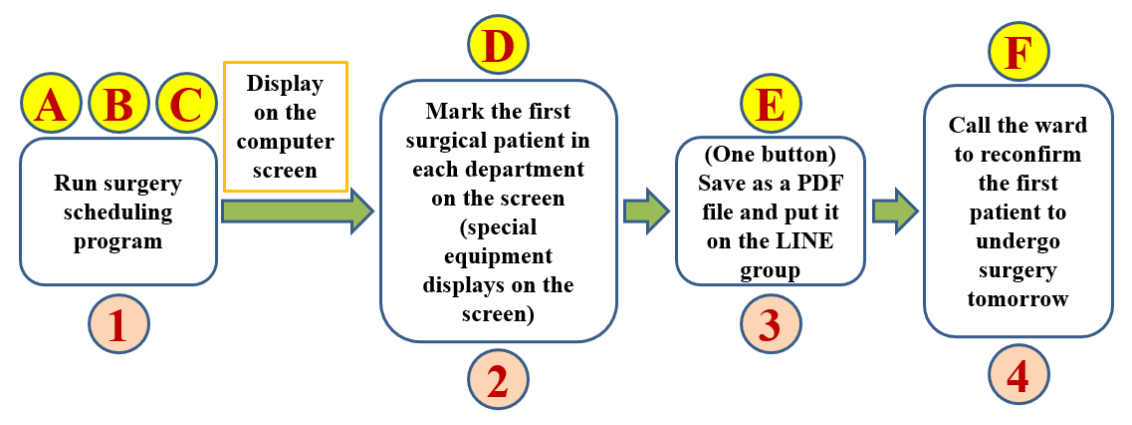
Future value stream map after the intervention of programmers in the Information Technology department of the hospital. Any program in the Information Technology department of our hospital can integrate our Excel macro into the hospital’s surgery information system to directly obtain data from the hospital’s database. This integration allows for the elimination and simplification of steps A, B, C, and E in the value stream map after improvement. Consequently, the future value stream map should further reduce movement and wait-time waste associated with file storage and program execution.

While satisfied with the outcomes (Figures 3 and 4) and confident in our adherence to lean principles and TPS methods, we must continue collecting numerical data and feedback from OR nurses using the Excel macro and reports daily. This evaluation is crucial for understanding their perceptions and confirming the enhancements’ effectiveness. Despite most nurses recognizing time savings in surgical scheduling, 4 out of 19 (21%) find the process not easier than before, and 3 out of 19 (16%) feel it does not meet their needs, indicating areas for further refinement. As Ohno [5] emphasized, if we know that we should improve things for the better, we must continue improving them until this task has been completed. This kind of persistence is the so-called soul of improvement [5]. The current VSM execution time of 261 seconds suggests the potential for further streamlining by integrating the macro directly into the hospital’s surgery information system, potentially eliminating steps A, B, C, and E to simplify future VSM and reduce waste associated with movement and wait times, as projected in Figure 5.

Taiichi Ohno, the founder of the TPS, emphasized that its main goal was to reduce costs to sustain operations during tough economic times like the oil crisis [5]. He believed that eliminating waste is essential for solving problems and reducing costs. Ohno also advocated for valuing equipment based on productivity rather than age and using existing resources instead of seeking costly new ones [5,6]. Similarly, Womack and Jones [4] argued against excessive spending to enhance business efficiency. In our project, we used the cost-free, built-in VBA of Excel, leveraging its capabilities to streamline our surgical scheduling system without new expenses. By optimizing existing tools and adhering to the principles of Ohno [5] and Womack and Jones [4], we effectively minimized waste and improved system efficiency.

### Algorithm Versus VSM

Algorithms are vital in computational thinking and are fundamental to problem-solving in computer programming. They detail step-by-step processes for addressing challenges in computation, information processing, and graphics [10,19-21]. Similarly, VSM offers structured processes for problem-solving, as shown in Figures 2 and 4. Both algorithms and VSMs proceed sequentially, with each step essential for problem resolution. Despite their application in different domains, their thinking processes and documentation methods are akin. Applying VSM creation methods to algorithms has proven beneficial based on our practical experience.

In the future, integrating TPS’s autonomation principle could enhance our system further. This involves giving machines human-like intelligence to stop operations when defects or anomalies are detected [4-7]. For example, if our system identifies a doctor’s scheduled operating hours exceeding hospital regulations, it should alert the operating director and chief nursing officer automatically. This automated notification feature mirrors Toyota’s autonomation process and is crucial for our system’s advancement.

### Limitations

This paper has some limitations. First, there was no further improvement in step F (Figure 4) to “call the ward to reconfirm the first patient to undergo surgery tomorrow” due to the need to comply with hospital regulations. There may be room for improvement in the future using digital signatures and 2-factor or 3-factor authentication systems, such as those used by banks. Second, to execute the macro in Excel, the security level of the computer must be lowered, which increases the chance of computer viruses invading the whole hospital information system. The use of a macro is not ideal. Third, this macro may fail when the Excel version is updated again. A nonprofessional programmer such as us may require several months to fix this issue.

Certain discerning individuals may indeed question whether the stated limitations will impact the sustainability of our changes. After extensive deliberations among our team, we recognize that some limitations might pose temporary challenges. However, given the relentless advancement of human society and IT, combined with our unwavering commitment to continuous improvement, we remain confident that these challenges can be addressed and overcome in due time. The primary limitation, as outlined, pertains to the current patient identification process within the hospital system. This process is foundational for ensuring patient safety and safeguarding patient privacy. Presently, identification involves manual phone calls to confirm a patient’s primary physician, inpatient bed number, essential details, time of transfer to the OR, and other relevant data. This is time-consuming. Although currently in our hospital, both doctors and nurses have secure digital authentication methods developed with national resources and efforts, a secure digital authentication method for patient identification has not yet been established. However, envision a future where patient identification becomes digitized, which would significantly expedite this process. With the rapid advancements in computer IT, especially the capabilities of artificial intelligence, and growing investments in the digital certification of national identities, we are optimistic that a robust digital identification system that guarantees both information security and patient privacy will emerge [22-24]. This, in turn, would address this limitation. The second and third limitations relate to the design and functionality of our surgical scheduling system, which, as of now, has not been prioritized by the hospital’s senior management. We are hopeful that as they recognize the system’s importance and involve the hospital’s IT staff, both these issues will be addressed concurrently. Meanwhile, our immediate responsibility is to diligently continue refining the surgical scheduling system we have developed.

### Conclusion

Lean thinking and Toyota’s methods have proven invaluable in revamping our surgical scheduling system. These methods have not only provided us with efficient tools but also instilled an effective mindset for problem-solving. This journey led to the creation of a cost-efficient, user-friendly surgical scheduling system, emphasizing the potential of lean principles in the hospital surgery information system.

## Appendix

Multimedia Appendix 1 The time spent (measured in seconds) on each action based on the value stream map before improvement.

Multimedia Appendix 2 The time spent (measured in seconds) on each action based on the value stream map after improvement.

Multimedia Appendix 3 Questionnaire survey after using the process before and after improvement.

Multimedia Appendix 4 Scope of human research cases exempt from review by the ethics committee.

## Abbreviations

OR: operating room
TPS: Toyota Production System
VBA: Visual Basic for Applications
VSM: value stream map

## Data Availability

The data sets analyzed during this study are available from the corresponding author upon reasonable request.
