# Supplementary material for: Developing a Cost-Effective Surgical Scheduling System Applying Lean Thinking and Toyota’s Methods for Surgery-Related Big Data for Improved Data Use in Hospitals: User-Centered Design Approach
Source: JMIR Form Res. 2024 May 24;8:e52185. doi: 10.2196/52185 (PMC11161709; doi:10.2196/52185)
Supplement: Multimedia Appendix 1 [file formative_v8i1e52185_app1.docx]

**Table S1.** The time spent (measured in seconds) on each action based on the value stream map before improvement.

| Time spent  Nurse  number | Time spent requesting the operation scheduling query system to output a report | Time spent printing out original data (1. Find and press the print button 2. Wait for data to print 3. Move to obtain printed data) | Time spent marking the first surgical patient in each department | Time spent indicating the special equipment that must be used | Average time spent calling the ward to reconfirm the first patient to undergo surgery tomorrow | Total amount of time spent |
| --- | --- | --- | --- | --- | --- | --- |
| 1 | 42 | 27 | 13 | 25 | 98 | 205 |
| 2 | 30 | 22 | 17 | 40 | 140 | 249 |
| 3 | 66 | 23 | 15 | 50 | 121 | 275 |
| 4 | 30 | 29 | 34 | 84 | 197 | 374 |
| 5 | 34 | 25 | 20 | 39 | 200 | 318 |
| 6 | 18 | 21 | 15 | 20 | 174 | 248 |
| 7 | 40 | 17 | 14 | 31 | 156 | 258 |
| 8 | 21 | 13 | 11 | 23 | 107 | 175 |
| 9 | 38 | 14 | 18 | 32 | 198 | 300 |
| 10 | 33 | 35 | 46 | 36 | 200 | 350 |
| 11 | 35 | 26 | 62 | 53 | 182 | 358 |
| 12 | 20 | 27 | 48 | 56 | 180 | 331 |
| 13 | 15 | 22 | 45 | 52 | 154 | 288 |
| 14 | 30 | 37 | 47 | 45 | 187 | 346 |
| 15 | 35 | 33 | 53 | 51 | 207 | 379 |
| 16 | 45 | 36 | 35 | 60 | 196 | 372 |
| 17 | 18 | 29 | 19 | 34 | 175 | 275 |
| 18 | 24 | 26 | 17 | 53 | 186 | 306 |
| 19 | 37 | 35 | 24 | 47 | 172 | 315 |
| *Mean* | 32 | 26 | 29 | 44 | 170 | 301 |
