# Supplementary material for: Developing a Cost-Effective Surgical Scheduling System Applying Lean Thinking and Toyota’s Methods for Surgery-Related Big Data for Improved Data Use in Hospitals: User-Centered Design Approach
Source: JMIR Form Res. 2024 May 24;8:e52185. doi: 10.2196/52185 (PMC11161709; doi:10.2196/52185)
Supplement: Multimedia Appendix 2 [file formative_v8i1e52185_app2.docx]

**Table S2.** The time spent (measured in seconds) on each action based on the value stream map after improvement.

| Time spent  Nurse  number | Time spent requesting the operation scheduling query system to output a report | Time spent saving original data as a file (Excel format) | Time spent finding and running the Macro program | Time spent marking the first surgical patient in each department on the screen | Time spent saving as a .pdf file and putting it on the Line group | Average time spent calling the ward to reconfirm the first patient to undergo surgery tomorrow | Total amount of time spent |
| --- | --- | --- | --- | --- | --- | --- | --- |
| 1 | 38 | 11 | 7 | 6 | 50 | 188 | 300 |
| 2 | 20 | 10 | 6 | 4 | 30 | 201 | 271 |
| 3 | 61 | 15 | 12 | 5 | 54 | 142 | 289 |
| 4 | 33 | 21 | 9 | 14 | 60 | 179 | 316 |
| 5 | 36 | 17 | 15 | 10 | 32 | 134 | 244 |
| 6 | 15 | 20 | 4 | 10 | 20 | 192 | 261 |
| 7 | 40 | 12 | 3 | 6 | 30 | 126 | 217 |
| 8 | 20 | 23 | 5 | 5 | 20 | 155 | 228 |
| 9 | 40 | 13 | 5 | 10 | 40 | 200 | 308 |
| 10 | 31 | 20 | 8 | 15 | 52 | 183 | 309 |
| 11 | 35 | 18 | 18 | 15 | 38 | 182 | 306 |
| 12 | 18 | 12 | 4 | 6 | 12 | 153 | 205 |
| 13 | 17 | 10 | 3 | 5 | 15 | 109 | 159 |
| 14 | 30 | 15 | 10 | 15 | 20 | 187 | 277 |
| 15 | 38 | 20 | 3 | 10 | 25 | 175 | 271 |
| 16 | 21 | 8 | 4 | 5 | 20 | 198 | 256 |
| 17 | 26 | 19 | 5 | 13 | 15 | 184 | 262 |
| 18 | 31 | 15 | 7 | 13 | 28 | 207 | 301 |
| 19 | 28 | 12 | 5 | 10 | 18 | 112 | 185 |
| *Mean* | 30 | 15 | 7 | 9 | 30 | 169 | 261 |
