# Supplementary material for: Developing a Cost-Effective Surgical Scheduling System Applying Lean Thinking and Toyota’s Methods for Surgery-Related Big Data for Improved Data Use in Hospitals: User-Centered Design Approach
Source: JMIR Form Res. 2024 May 24;8:e52185. doi: 10.2196/52185 (PMC11161709; doi:10.2196/52185)
Supplement: Multimedia Appendix 3 [file formative_v8i1e52185_app3.docx]

**Table S3.** Questionnaire survey after using the process before and after improvement.

| Question  Nurse  number | The process after improvement saves my time  (Yes or No) | The process after improvement is easier for me to use  (Yes or No) | The process after improvement meets my needs  (Yes or No) | If you could decide, you would choose the process after improvement  (Yes or No) |
| --- | --- | --- | --- | --- |
| 1 | Yes | Yes | Yes | Yes |
| 2 | Yes | Yes | Yes | Yes |
| 3 | Yes | No | Yes | Yes |
| 4 | Yes | Yes | Yes | Yes |
| 5 | Yes | Yes | No | No |
| 6 | Yes | Yes | Yes | Yes |
| 7 | Yes | No | No | No |
| 8 | Yes | No | No | No |
| 9 | Yes | Yes | Yes | Yes |
| 10 | Yes | Yes | Yes | Yes |
| 11 | Yes | Yes | Yes | Yes |
| 12 | Yes | Yes | Yes | Yes |
| 13 | Yes | Yes | Yes | Yes |
| 14 | Yes | Yes | Yes | Yes |
| 15 | Yes | Yes | Yes | Yes |
| 16 | Yes | No | Yes | Yes |
| 17 | Yes | Yes | Yes | Yes |
| 18 | Yes | Yes | Yes | Yes |
| 19 | Yes | Yes | Yes | Yes |
| Total number of “Yes” | 19  (100%) | 15  (79%) | 16  (84%) | 16  (84%) |
