# Supplementary material for: Developing a Cost-Effective Surgical Scheduling System Applying Lean Thinking and Toyota’s Methods for Surgery-Related Big Data for Improved Data Use in Hospitals: User-Centered Design Approach
Source: JMIR Form Res. 2024 May 24;8:e52185. doi: 10.2196/52185 (PMC11161709; doi:10.2196/52185)
Supplement: Multimedia Appendix 4 [file formative_v8i1e52185_app4.doc]

**Scope of Human Research Cases Exempt from Review by the Ethics Committee**

July 5, 2012

**Announcement No. 1010265075 by the Department of Health, Medical Affairs**

Research cases that do not involve minors, detainees, indigenous people, pregnant women, persons with disabilities, psychiatric patients, and other individuals determined by the review committee to be under undue coercion or unable to make decisions freely, and that meet one of the following conditions, may be exempt from submission to the Ethics Review Committee or may receive an exemption certificate issued by the committee:

1. Research conducted in public settings that is anonymous, non-interactive, and non-interventional, and where information collected does not identify specific individuals.
2. Use of legally publicly available information that is in accordance with the purpose for which it was made publicly known.
3. Public policy effectiveness research conducted by government agencies or by professional institutions commissioned by them as part of their statutory duties.
4. Research on educational assessment or testing, teaching techniques, or effectiveness evaluations conducted within a typical educational environment.
5. Research projects that pose minimal risk, where the risk to the participants is no greater than for non-participants, evaluated by the Ethics Review Committee as exempt and issued an exemption certificate.

得免倫理審查委員會審查之人體研究案件範圍

101年7月5日

衛署醫字第1010265075號公告

研究案件非以未成年人、收容人、原住民、孕婦、身心障礙、精神病患及其他經審查會訂定或判斷受不當脅迫或無法以自由意願做決定者為研究對象，且符合下列情形之一，得免送倫理審查委員會審查或由倫理審查委員會核發免審證明：

1. 於公開場合進行之非記名、非互動且非介入性之研究，且無從自蒐集之資訊辨識特定之個人。
2. 使用已合法公開週知之資訊，且資訊之使用符合其公開週知之目的。
3. 公務機關執行法定職務，自行或委託專業機構進行之公共政策成效評估研究。
4. 於一般教學環境中進行之教育評量或測試、教學技巧或成效評估之研究。
5. 研究計畫屬最低風險，且其研究對象所遭受之風險不高於未參加該研究者，經倫理審查委員會評估得免審查並核發免審證明。

前項最低風險，係指研究對象所遭受之危害或不適的機率或強度，不高於日常生活中遭受的危害或不適。
